# A survey of ambulance clinicians’ perceptions of recording and communicating patient information electronically

**DOI:** 10.29045/14784726.2021.6.6.1.1

**Published:** 2021-05-01

**Authors:** Jack William Barrett, Pete Eaton-Williams, Craig ED Mortimer, Victoria FP Land, Julia Williams

**Affiliations:** South East Coast Ambulance Service NHS Foundation Trust; South East Coast Ambulance Service NHS Foundation Trust; South East Coast Ambulance Service NHS Foundation Trust; South East Coast Ambulance Service NHS Foundation Trust; South East Coast Ambulance Service NHS Foundation Trust; University of Hertfordshire

**Keywords:** ambulance, EMS, ePCR, healthcare records, technology

## Abstract

**Objective:**

Ambulance services are evolving from use of paper-based recording of patient information to electronic platforms and the impact of this change has yet to be fully explored. The aim of this study is to explore how the introduction of a system permitting electronic information capture and its subsequent sharing were perceived by the ambulance clinicians using it.

**Methods:**

An online questionnaire was designed based upon the technology acceptance model and distributed throughout one ambulance service in the south east of England. Closed-ended questions with Likert scales were used to collect data from patient-facing staff who use an online community falls and diabetic referral platform or an electronic messaging system to update GPs following a patient encounter.

**Results:**

There were 273 responses from ambulance clinicians. Most participants agreed that they used tablet computers and smartphones to make their life easier (85% and 86%, respectively). Most participants felt that referring patients to a community falls or diabetic team electronically was an efficient use of their time (81% and 81%, respectively) and many believed that these systems improved the communication of confidential patient information. GP summaries were perceived as increasing time spent on scene but most participants (89%) believed they enabled collaborative working. Overall, collecting and sharing patient information electronically was perceived by most participants as beneficial to their practice.

**Conclusion:**

In this study, the ability to electronically refer patients to community services and share patient encounters with the GP was predominantly perceived as both safe for patients and an effective use of the participants’ clinical time. However, there is often still a need to communicate to GPs in real time, demonstrating that technology could complement, rather than replace, how clinicians communicate.

## Introduction

Between 2015 and 2016, NHS ambulance trusts received 10.7 million 999 or 111 calls, of which 6.6 million resulted in face-to-face patient contact (National Audit Office, 2017). Communication of patient information between healthcare professionals and patient services in the NHS is vital to patient care and safety. Historically, emergency medical service (EMS) clinicians have shared patient information through paper clinical records, telephone or face-to-face conversations. Government-led initiatives have seen ambulance services adopt technology in the workplace so that patient information can be recorded and shared electronically (Department of Health, 2016; NHS England, 2014). The implementation of iPads and Toughbooks in ambulance services in the UK has been challenging for ambulance trusts but has been achieved with reasonable success and acceptance from staff (Porter et al., 2019).

The adoption of recording patient information electronically in ambulance services has allowed clinicians to send information to the patient’s GP or to refer patients to other services electronically instead of speaking to the receiving healthcare professional directly or sending a facsimile. This evolution reduces the need to spend time on the telephone contacting other healthcare services and allows secure transmission of confidential patient information via a secure communication network.

There is a paucity of research on ambulance services’ use of electronic patient reporting systems (Porter et al., 2019; Wood et al., 2015). However, electronic patient care records (ePCRs) have been used by clinicians in hospital for the last two decades and have been linked to improving the quality and efficiency of healthcare (Chaudhry et al., 2006), with recording patient information at the bedside being identified as efficient use of nursing time (Poissant et al., 2005). However, there have also been some limitations noted with the use of ePCRs. It has been reported by nurses and midwives that ePCRs do not improve clinical practice or patient care, citing errors in data entry and retrieval (Darbyshire, 2004). Inappropriate organisation implementation can disturb previously efficient work routines and cause a breakdown in communication, contributing to increasing workloads rather than reducing them (Ash et al., 2004). The success and optimisation of ePCRs in healthcare systems are down to the quality of the hardware and software implemented by the organisation (Aarts & Berg, 2006) and the receptiveness of the end user – the clinician – to using the technology to its full potential (de Veer & Francke, 2010).

The aim of this study is to explore how a system permitting electronic information capture and communication in one ambulance service is perceived by the clinical EMS staff using it.

## Methods

### Study setting

South East Coast Ambulance Service NHS Foundation Trust (SECAmb) provides unscheduled emergency and urgent care across the south east of England, covering 3600 square miles and a population of 4.2 million people, and receiving approximately 862,000 emergency calls a year. SECAmb employs approximately 2000 members of staff responsible for responding to emergency and urgent calls predominantly on double-crewed ambulances. SECAmb currently has two electronic patient recording platforms: (1) the ePCR, which is used to capture and collect patient information for every face-to-face contact and replaces the paper patient care record, and (2) the Intelligence Based Information System (IBIS), a database of patient care plans that also enables staff to electronically refer patients to community falls and diabetic teams or send a written summary of a patient encounter to the patient’s GP. The IBIS system aims to improve interaction between the ambulance service, community providers and primary care to reduce unnecessary ambulance conveyance to hospital and has been in place since 2012. Originally, a clinician would telephone a non-clinician at the emergency operations centre and would pass a referral on to the appropriate team. This system then moved to iPads in November 2017 which allowed clinicians to make referrals directly or complete a GP summary notification at the patient’s side.

All SECAmb staff that work on ambulances are issued with, or have access to, an iPad enabling access to the ePCR and IBIS platforms. Due to a delay in the implementation of ePCRs in the Trust at the time of this study, staff did not have enough experience with the ePCR platform, so it was decided that data collection would focus on staff perceptions and experiences of using the IBIS platform. IBIS is linked to 12 community falls teams covering the Trust’s geographical area and 781 GP sites across 31 clinical commissioning groups.

### Questionnaire design

A questionnaire was designed to gain an understanding of staff perceptions of recording and sharing patient information electronically. The questionnaire was developed with reference to the technology acceptance model (TAM) (Davis et al., 1989; de Veer & Francke, 2010), which explores variables that contribute to whether a piece of technology will be accepted by the user (Ma & Liu, 2004) and has undergone several iterations to improve information technology implementation (Venkatesh & Bala, 2008). The TAM proposes that an intention to accept technology is determined by three domains:

1. Attitude: the individual’s response to new technology.
2. Perceived usefulness: the individual’s perception that adopting a new technology will be superior to current practice.
3. Perceived ease of use: that the implementation of new technology will be comparatively easy without excessive use of resources.

The literature surrounding the TAM is well established, supporting the model’s reliability and robustness in various information system environments. Its validity makes it ideal to explore ePCR implementation in the emergency and urgent care sector (de Veer & Francke, 2010). Furthermore, the TAM can be applied to individuals at all levels of computer literacy (Lai & Li, 2005; Yu et al., 2005), of either sex and any age (Lai & Li, 2005), and to those working in western cultures (McCoy et al., 2005; Straub et al., 1997).

A sample questionnaire can be found in Supplementary 1 and is based on previous work conducted in other healthcare settings utilising the TAM (de Veer & Francke, 2010) and adapted for use in this study. The questionnaire was piloted on a small number of staff and changes made based on feedback, which was predominantly about question clarity and avoiding topic overlap. Once completed, the questionnaire was uploaded onto an online platform ready for participant recruitment (Qualtrics, Provo, UT).

### Participant recruitment and eligibility

Non-probability purposive volunteer sampling was utilised in this study. A series of advertisements was placed in Trust bulletins, emails and on staff notice boards advertising the study and directing staff to the online questionnaire either via a secure URL hyperlink or QR code to scan. On arrival to the site a participant information sheet was available for review followed by a consent form. Participants were eligible to enrol on the study if they were a SECAmb ambulance clinician who had completed at least one of the following: GP summary notification, hypoglycaemic episode notification and/or community falls referral. Participants were excluded if access to an IBIS record had been for the purposes of audit, service evaluation, quality assurance/improvement or any other reason not directly related to continuing patient care.

All clinical grades are eligible to complete falls and hypoglycaemia notifications, while paramedics are expected to send GP summary notifications. However, non-registered healthcare professionals can scribe for the paramedic who would then review the inputted information and submit it.

A target sample size of at least 250 was set by the study team; this was based on previous questionnaire-based studies the Trust had delivered.

### Data collection

The questionnaire collected anonymous demographic data, experience and acceptance of using technology in general. Participants were then asked to identify which functions of the IBIS platform they had used, and the questionnaire then populated questions relevant to the participants’ responses, which encapsulated all three elements of the TAM model; 5-point Likert scales were used to collect participant responses.

The questionnaire was opened to SECAmb staff on 19 February 2019 and closed on 2 March 2020. The study was opened for an extended period due to attempts made by the study team to collect comparative data from healthcare professionals receiving referrals; however, this endeavour was ultimately unsuccessful. During this period there were no changes in how the IBIS platform worked nor how it was accessed through the iPad, thus the user experience did not differ through the study period.

Data were collated on Microsoft^®^ Excel 2019 (Microsoft Corporation, Version 16). Descriptive statistics are used to present the findings; proportions of the total number of responses are presented as percentages (%). Responses are clustered into their respective themes according to the TAM model described above, and participants’ overall responses then interpreted as positive, neutral or negative based on the proportion of responses given to the question. To quantify this, where a question had a response of 50% or more, this response was deemed to be the prominent view. If no response was greater than 40% it was considered that there was no difference between responses. These were adjudicated by the lead author and second author and reported on accordingly.

## Results

Of a total of 291 responses collected, following review 18 participants were excluded due to consenting to the study but not answering questions relating to their experience or acceptance of technology nor the GP, falls or diabetic referral questions. Responses from 273 SECAmb participants were included in the analysis, a response rate of approximately 14%. However, some sections were still not fully completed by these participants. Questions where responses were less than the total are reported on accordingly and percentages are calculated from the total responses to individual questions.

Table 1 outlines the demographics of the respondents involved in the SECAmb questionnaire. A total of 266 (97.44%) answered questions related to the TAM, on general use of technology and acceptance of new technology. Most participants agreed that they used tablet computers and smartphones to make their life easier (n = 225/266, 85% and n = 228/265, 86%, respectively), with most agreeing that they enjoyed using tablet computers and smartphones (n = 203/266, 76% and n = 209/266, 79%, respectively). The majority felt that they could teach themselves how to use a new piece of technology in the workplace (n = 203/266, 76%); however, when asked if they would need help to learn about a new piece of equipment, opinion was split, with 32% (n = 86/266) indicating they would need help and 43% (n = 115/266) indicating they would not.

**Table 1.** Demographic descriptors of SECAmb questionnaire responses.

	Total (273)
Sex:	**n (%)**
Male	161 (59)
Female	105 (38)
Undisclosed	7 (3)
Age:	**n (%)**
18–24	49 (18)
25–29	54 (20)
30–34	41 (15)
35–39	31 (11)
40–44	37 (14)
45–49	31 (11)
50–54	14 (5)
55–59	12 (4)
60–64	3 (1)
Undisclosed	1 (0)
Job title:	**n (%)**
Critical care paramedic	8 (3)
Emergency care support worker	31 (11)
Newly qualified paramedic	29 (11)
Paramedic	120 (44)
Paramedic practitioner	24 (9)
Student paramedic	19 (7)
Undisclosed	3 (1)
Team leader/senior manager	8 (3)
Technician	31 (11)
Years as a healthcare professional:	**n (%)**
< 1	42 (15)
1	16 (6)
2	23 (8)
3	16 (6)
4	25 (9)
5	20 (7)
6	6 (2)
7	10 (4)
8	9 (3)
9	9 (3)
10	12 (4)
11	8 (3)
12+	76 (28)
Undisclosed	1 (0)
IBIS function used	**n (%)**
Falls referral	250 (92)
General practitioner summary	214 (78)
Hypoglycaemic referral	129 (47)

IBIS = intelligence based information system.

### Staff perception of the IBIS falls referral platform

There were 250 participants who declared they had previously used an electronic falls referral, of which 233 (92%) completed the falls referral section of the questionnaire. Just under half of participants agreed (n = 114/232, 49%) they would find it difficult to make a referral without having the electronic platform available to them. Consequently, the majority agreed (n = 155/233, 67%) that because the platform was available to them more patients would be referred, with 75% (n = 174/232) suggesting that they currently use the platform on every appropriate patient. Most participants perceived that using the electronic referral platform was an efficient use of their time (n = 187/231, 81%) and 161 participants (69%) felt that the platform enabled collaborative working with the falls team.

When asked whether participants would prefer to speak to the falls team directly (via telephone) or send a referral electronically, 105/233 (45%) indicated they would prefer to send an electronic referral. Over half of the participants who used the falls referral platform (n = 129/233, 55%) felt that sending referrals electronically better protected confidential patient information, with a similar proportion (n = 125/232, 54%) believing that patient care was improved because of electronic referrals. Overall, participants agreed (n = 193/230, 84%) that having the electronic referral platform was a benefit to their role.

### Staff perception of the general practitioner summary platform

Of the 214 participants who identified that they had sent a GP summary, 204 (95%) completed the GP summary section of the questionnaire. Around a third of participants felt that the difficulty of their work would not increase if they did not have the GP summary function (n = 70/204, 34%), and a similar proportion (n = 81/204, 40%) did not feel it was necessary to send a GP summary following every patient interaction. However, 137/204 (67%) participants found the platform easy to use and 141/204 (69%) agreed that it addressed their need to communicate with the patient’s GP, with most participants (n = 185/204, 91%) agreeing it was an effective means to capture and share patient information.

Almost half of these participants (n = 90/203, 44%) neither agreed nor disagreed that confidential patient information was better protected on the GP summary platform compared to speaking directly to the GP via telephone; but a significant number (n = 87/204, 43%) indicated that they would prefer to speak directly to the GP. By sending the GP summary, 174/204 (85%) participants believed they were working collaboratively with the GP and a similar proportion (n = 181/204, 89%) believed that the GP would have a better understanding of the patient interaction. Most participants felt that they spent longer on scene (n = 123/203, 61%) when completing a GP summary, although most agreed (n = 139/203 68%) that this was an effective use of their time. Overall, participants liked the ability to send the GP summary (n = 182/204, 89%) and agreed it was useful to their job (n = 179/203, 88%).

### Staff perception of the hypoglycaemic referral platform

Of the 129 participants who stated they had previously completed an electronic diabetic referral, 122 (94.57%) completed this section of the questionnaire. Most participants (n = 56/122, 46%) agreed that it would be difficult to refer a patient without the electronic platform and most (n = 102/122, 84%) found it easy to use. Staff were divided on whether they preferred to speak to another healthcare professional rather then send the referral electronically (electronic referral n = 49/122, 40%; telephone, n = 37/122, 30%; no preference n = 36/122, 30%). However, staff believed it was safe to refer patients using the platform (n = 100/121, 83%) and perceived that confidential patient information was better protected when sending an electronic referral (n = 76/122, 62%). As a result of this system’s availability, participants perceived that more patients would be referred (n = 92/122, 75%) and that this was an efficient use of their time (n = 98/121, 81%). Overall, participants preferred the electronic referral platform (n = 103/122, 84%) and found it useful in their job (n = 111/122, 91%).

## Discussion

The findings from this questionnaire suggest that staff have a positive attitude towards new technology, and many can learn how to use it with little assistance. Ambulance staff responding to this questionnaire agreed that they used smartphones and tablet computers to make their life easier (86% and 85%, respectively) and were confident that they could teach themselves to use a new piece of technology (76%), suggesting ambulance clinicians are receptive to the introduction of technology within their workplace. However, a third of respondents (32%) indicated that they would need help to utilise new equipment, suggesting that employers need to be conscious of their implementation strategies.

The introduction of technology to record and share patient information is a significant transition within the ambulance service. Its success is dependent on both an effective implementation strategy and acceptance by the staff using it (de Veer & Francke, 2010). The TAM provides a useful means to measure how likely staff are to accept new technology in the workplace (Davis et al., 1989; de Veer & Francke, 2010) and its three domains of attitude, perceived usefulness and ease of use can help to understand whether a workforce can or would adopt technology into their practice. These findings may suggest that while staff are receptive to new technology in the workplace, ambulance trusts need to be supportive to all staff when introducing new technology to ensure end user uptake is as high as possible.

Staff reported that using an online referral platform increased their time on scene but perceived that this was an efficient use of their time. Staff indicated that having the ability to electronically refer patients to a community falls or diabetic team made the referral process easier for them. The NHS often uses time as a metric of efficiency in service delivery, and it has been proposed that technology has the ability to allow staff to be more efficient with their time at the patient’s side (Chaudhry et al., 2006). Ambulance clinicians have stated that with experience they become quicker at recording information electronically (Porter et al., 2019), but if staff perceive that technology extends the length of time it takes to complete a task, then this could deter them from engaging with it. Despite this concern, most staff in this study reported that using an electronic platform to share patient information was an effective use of their time, suggesting they value its perceived benefits and accepted this technology.

Staff found the electronic platform useful to their practice, indicating that they felt more appropriate referrals would be made and it enabled collaborative working. However, there are instances where a real-time direct conversation is preferred, supporting the idea that new technology should augment how ambulance service staff communicate with other healthcare professionals and not replace previous methods (Hertzum & Simonsen, 2008).

Patient confidentiality is a priority in healthcare systems (Wachter & Cassel, 2020) and many staff surveyed believed that patient information shared electronically is better protected than via telephone conversation. However, when sending a GP summary there was less perceived confidence in the electronic sending of a summary and many staff preferred to speak directly to the GP. The difference between staff attitudes towards electronic referral and a GP summary could be that referrals usually follow strict inclusion and exclusion criteria. Following a competent assessment by an ambulance clinician with the aid of decision tools, clinicians should be able to determine whether their patient is suitable for a referral to the community falls team without the need for a telephone discussion (Snooks et al., 2017). However, more complex community management plans may necessitate interactive discussion with the healthcare professional, such as the GP, who has prior knowledge of the patient and who may also be responsible for the implementation or subsequent review of those plans.

### Limitations

There are several limitations to this study that should be acknowledged. Firstly, this questionnaire used closed-ended questions, allowing participants to respond through Likert scales only. While this provides an assessment of staff perceptions on the use of technology to communicate patient information, it does not provide context to the answers given. There were some data missing; however, this was a small amount and unlikely to drastically change the findings from this study. Secondly, the questionnaire was hosted on an online platform; therefore it is likely that those who responded were more likely to use and enjoy the use of digital platforms and the perceptions of staff who do not enjoy using technology or find it difficult to use may be missed. This could mean technology acceptance in the ambulance service is misrepresented. Thirdly, a recognised limitation of the TAM, and consequently of this study, is that it cannot fully account for the effects of external variables, such as workplace culture, on the behavioural intentions of the population under scrutiny (Mathieson et al., 2001). Finally, this study represents the opinions of ambulance personnel sending electronic referrals. While the electronic referral platforms have been received positively by staff surveyed in this study, it is unknown and we were unable to ascertain whether this view is shared by those receiving the referrals.

## Conclusions

Ambulance clinicians need reliable systems that they have confidence in to securely record and communicate electronic patient information. The majority of staff participating in this study reported they enjoyed using technology and it makes their life easier. The ability to electronically refer patients to community services and share patient encounters with the GP is seen as both an effective use of clinical time and safe for patients. However, there still appear to be some incidents where staff want real-time discussion, highlighting that electronic communication currently complements rather than replaces traditional forms of communication. Future research should further explore staff perceptions of recording and sharing patient information electronically and consider the impact it has on both the patient experience and the receiving healthcare professionals that use this information.

## Acknowledgements

The authors would like to thank the SECAmb IBIS systems manager Stephanie Fisher for her support and for supplying the contact details for the partner primary care sites, and Joanna Gardner, Gerardine Power and Laura Walsh for their support in distributing promotional material and for recruitment into the study. A special thanks to the members of SECAmb staff who took time to review the first edition of the study questionnaire and provide feedback for its refinement.

## Author contributions

JB conceived the study, all authors contributed to the development of the research question and methodology. The questionnaire was developed by JB and PEW, with critique from JW, CM and VL. JB and VL screened the original questionnaire in its intended populations and JB, PEW and VL refined it before the study went live. Data collection and analysis were performed by JB with oversight from PEW. The manuscript was written by JB and all authors contributed to its edit before submission. JB acts as the guarantor for this article.

## Conflict of interest

JW is on the editorial board of the BPJ.

## Ethics

Ethical approval was sought, and a favourable opinion granted by the Health Research Authority (REC reference: 19/HRA/0766).

## Funding

This study was funded by the College of Paramedics’ Small Research Grant.
